# Biodegradable double-targeted PTX-mPEG-PLGA nanoparticles for ultrasound contrast enhanced imaging and antitumor therapy *in vitro*

**DOI:** 10.18632/oncotarget.13243

**Published:** 2016-11-09

**Authors:** Jing Ma, Ming Shen, Chang Song Xu, Ying Sun, You Rong Duan, Lian Fang Du

**Affiliations:** ^1^ Department of Ultrasound, Songjiang Hospital Affiliated to The First People's Hospital of Shanghai Jiao tong University, Shanghai 201600, China; ^2^ Department of Ultrasound, Shanghai First People's Hospital Affiliated to Shanghai Jiao tong University School of Medicine, Shanghai 200080, China; ^3^ Department of Ultrasound, Shanghai East Hospital Affiliated to Tong ji University, Shanghai 200120, China; ^4^ State Key Laboratory of Oncogenes and Related Genes, Shanghai Cancer Institute, Renji Hospital, School of Medicine, Shanghai Jiao Tong University, Shanghai 200032, P. R. China; ^5^ Huai'an First People's Hospital, Nanjing Medical University, Jiangsu 223001, China

**Keywords:** double-targeted, nanoparticles, antitumor, ultrasound contrast enhanced imaging, pancreatic cancer

## Abstract

A porous-structure nano-scale ultrasound contrast agent (UCA) was made of monomethoxypoly (ethylene glycol)-poly (lactic-co-glycolic acid) (mPEG-PLGA), and modified by double-targeted antibody: anti-carcinoembryonic antigen (CEA) and anti-carbohydrate antigen 19-9 (CA19-9), as a double-targeted nanoparticles (NPs). Anti-tumor drug paclitaxel (PTX) was encapsulated in the double-targeted nanoparticles (NPs). The morphor and release curve were characterized. We verified a certain anticancer effect of PTX-NPs through cytotoxicity experiments. The cell uptake result showed much more NPs may be facilitated to ingress the cells or tissues with ultrasound (US) or ultrasound targeted microbubble destruction (UTMD) transient sonoporation *in vitro*. Ultrasound contrast-enhanced images *in vitro* and *in vivo* were investigated. Compared with SonoVue, the NPs prolonged imaging time in rabbit kidneys and tumor of nude mice, which make it possible to further enhance anti-tumor effects by extending retention time in the tumor region. The novel double-targeted NPs with the function of ultrasound contrast enhanced imaging and anti-tumor therapy can be a promising way in clinic.

## INTRODUCTION

As a kind of safe biodegradable organic polymeric material approved by the FDA for human medical use, poly (lactic-co-glycolic acid) modified with mPEG (mPEG-PLGA) has been used in drug delivery system [1]. CA19-9 and CEA are specific markers highly expressed on the surface of pancreatic carcinoma cells. Particularly, the CA19-9 is rarely expressed or detected in normal tissue cells [2, 3]. We carried out some concerned experiments that the PTX-mPEG-PLGA NPs graft-modified by CA19-9 and CEA antibodies may specifically recognize and adhere to the surface of pancreatic cancer cells or tissues, sustained-release PTX to kill tumor cells or inhibition its multiply. Besides, as a good physical promoting way, US or UTMD may facilitate loading drugs and gene nanoparticles uptaken by goal cells or tissues, such as tumors, eyes, skeletal muscle, heart and bone marrow stem cells without apparent tissue damage, and enhance drug or gene release in situ [4–10]. Thereby, we performed relevant experiments and found that much more double-targeted PTX-mPEG-PLGA NPs were transferred into pancreatic cancer cells or tissues with US or UTMD, compared with those without US.

Although many researches on targeted drug-loaded NPs delivery for tumor therapy have been done [11–15], the study on US real-time monitoring at the same time of anti-tumor treatment was rarely been reported.

We prepared double-targeted PTX-mPEG-PLGA NPs with double emulsion method. The inner water phase was removed by lyophilization and got sphericals with porous structure which may provide a basis for the excellent US scatterings, these nanoparticles, as a kind of novel US nano-UCAs, compared with SonoVue that were widely used for the clinical assessment of various diseases, some similar good contrast enhanced imagines with double-targeted PTX-mPEG-PLGA NPs are showed *in vitro* and *in vivo*. Our aim is to introduce this novel double-targeted nano-UCA with the integration of ultrasound contrast enhanced imaging and therapy.

## RESULTS

### Fabrication and characterization of double-targeted NPs

**Figure 1:**
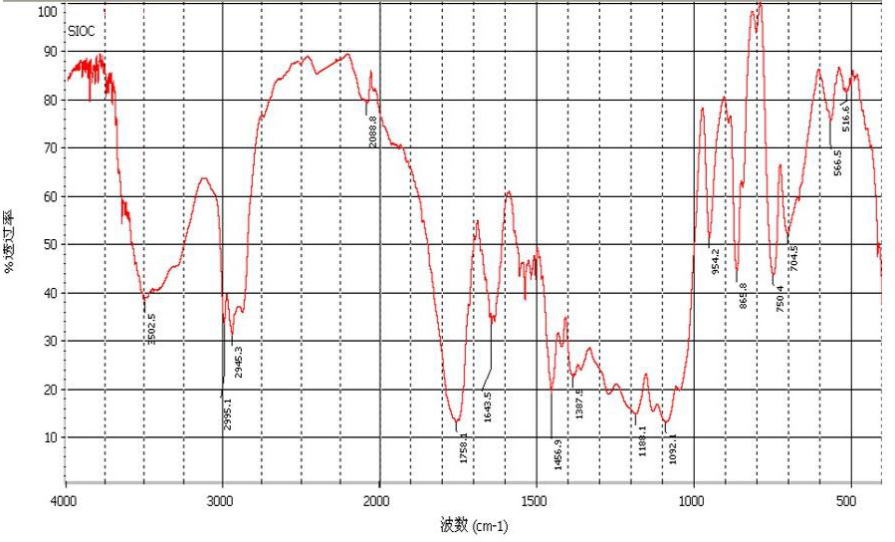
Infrared spectrum of double-targeted NPs

In infrared spectrum of double-targeted NPs (Figure 1), there were the ester bond peak in PLGA (1757cm^−1^), the amide bond peak in anti-CEA and anti-CA19-9 antibody (1620cm^−1^) and the carbon-oxygen bond peak in mPEG (1034cm^−1^). That proved the double-targeted NPs was made up of mPEG-PLGA modified by targeted-antibody (anti-CEA and anti-CA-19-9 antibody).

**Figure 2:**
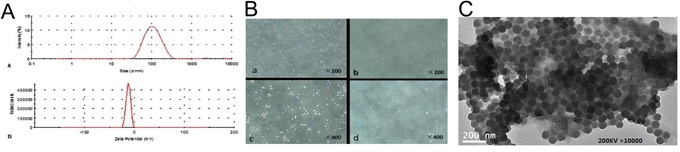
morphology of double-targeted NPs **A.** Size distributions and zeta potential distribution of double-targeted NPs. **B.** SonoVue (a, c) and NPs (b, d) degassed aqueous observed by LM (×200, ×400). **C.** TEM of double-targeted NPs.

The peak and average size of double-targeted NPs were respectively 136.3±5.2 nm and 88.6±3.5 nm (Figure 2A). The zeta potential of the NPs was −13.4±1.5 mv (Figure 2B). Under light microscopy, a large quantities of microbubbles of SonoVue degassed aqueous were viewed, much more NPs were rarely seen, which may demonstrate that the size of this kind of novel NPs was much less than that of SonoVue microbubble (Figure 2B). The morphology of NPs viewed by TEM was spherical with excellent dispersion and no aggregation (Figure 2C).

**Figure 3:**
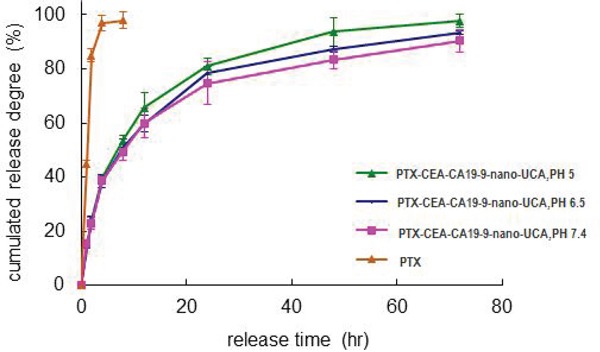
The PTX release from double-targeted NPs in different pH

The entrapment efficiency and loading drug efficiency of double-targeted NPs were separately 91.32±3.25% and 2.666±0.092%. About 97.57% of PTX was released during 4 hours from PTX solution. Whereas the release from NPs was slow and sustained as about 40% of the total was released in 4 hours and about 97.83% of the total was completely released in 72 hours. The PTX release rates from NPs at PH 5.0, 6.5, 7.4 condition were similar, which predicted the drug release rates *in vivo* were under no influence of pH (Figure 3). The properties of slow and sustained release made it possible for anti-cancer drug to keep effect continuously and efficiently.

### Optimal US or UTMD condition

**Figure 4:**
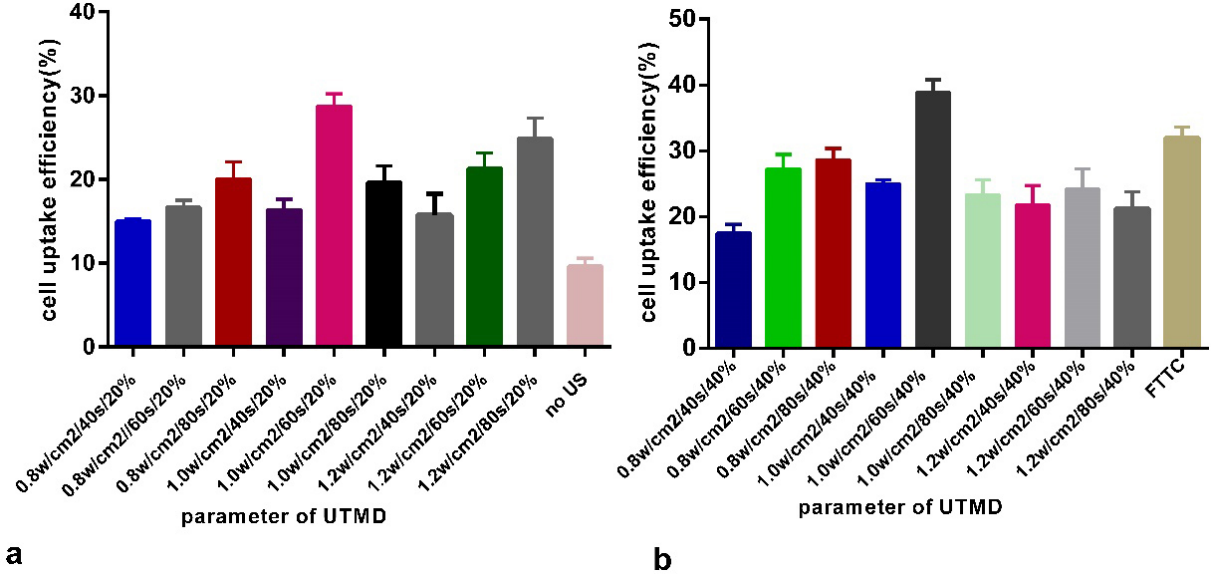
Optimal US or UTMD condition

The maximum cell uptake efficiency was 39.67±2.45%. The selected optimal UTMD condition was: power, 1 W/cm^2^, exposure time, 60 sec, SonoVue volume ratio, 2:5. Under the same optimal US condition, the cell uptake efficiency at 2:5 SonoVue volume ratio was higher than the one at 1:5 ratio, more amplified cavitational effects may be a major cause. (Figure 4).

### Cell uptake

Greater and stronger red fluorescence was observed in the cytoplasm of CFAPC-1 cell at 2 h incubation of the RhB double-targeted NPs than RhB-PLGA-mPEG NPs, which demonstrated good cell targeting of the double-targeted NPs.

**Figure 5:**
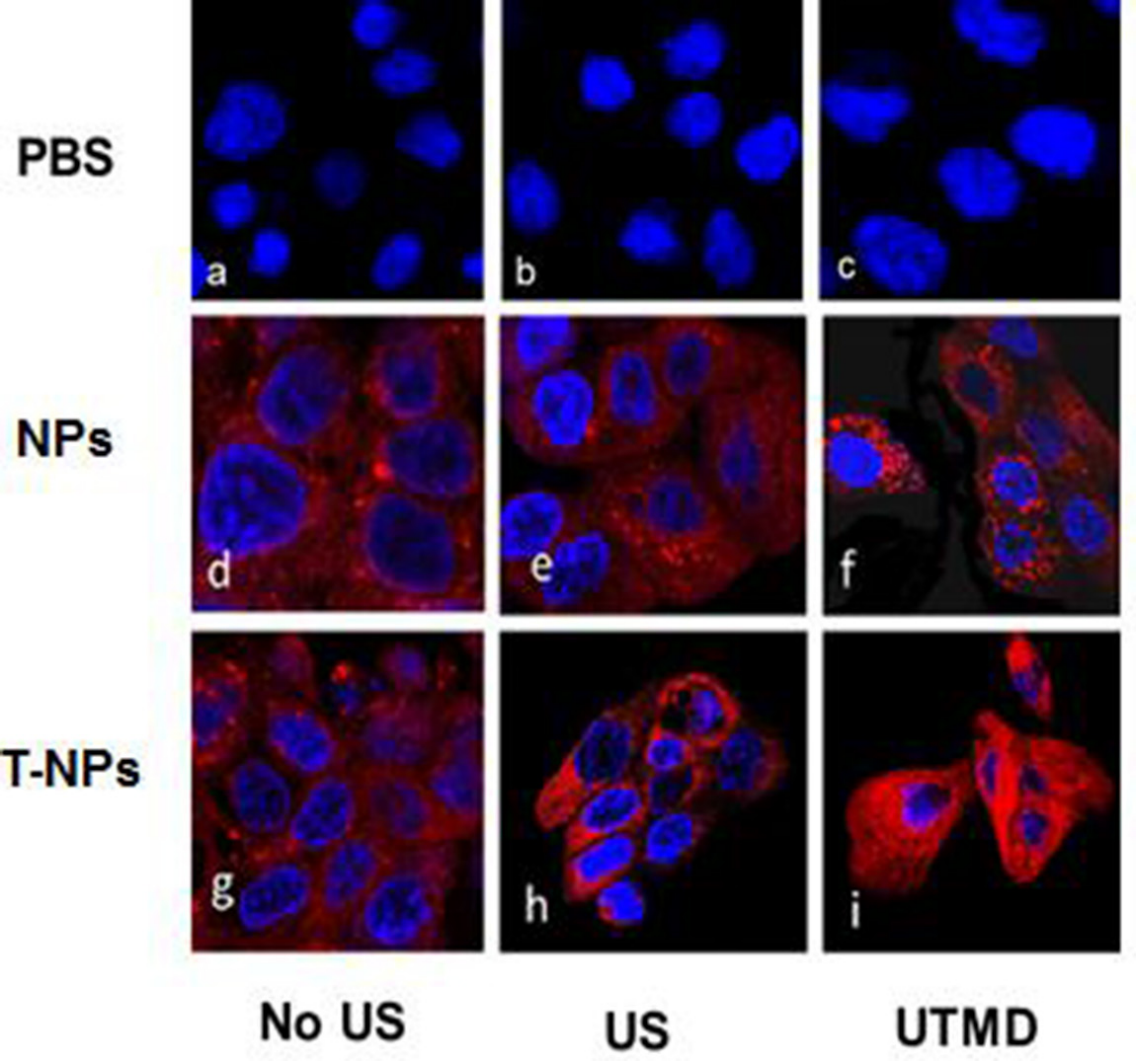
CLSM images of AFPC-1 cells after a 2 h incubation with the nanoparticles (NPs) with US or UTMD DAPI were blue fluorescent nuclear staining and red fluorescence was from the RhB.

Much stronger fluorescence was respectively observed in CFAPC-1 cells under the administration of US than no US in both of the two NPs. Greater fluorescence was observed in CFAPC-1 cells under the administration of UTMD than US, which manifested US may facilitate much more NPs into cells, while UTMD have greater impact on cells than US (Figure 5). Red fluorescence was found in the cytoplasm of CFAPC-1 cell, not in the nucleus, which illustrated that it was possible for NPs to penetrate the gap of cell membrane but it was too difficult for them to penetrate the nuclear membrane to the inner of nuclear, even with US and UTMD.

### Cytotoxicity of double-targeted NPs

**Figure 6:**
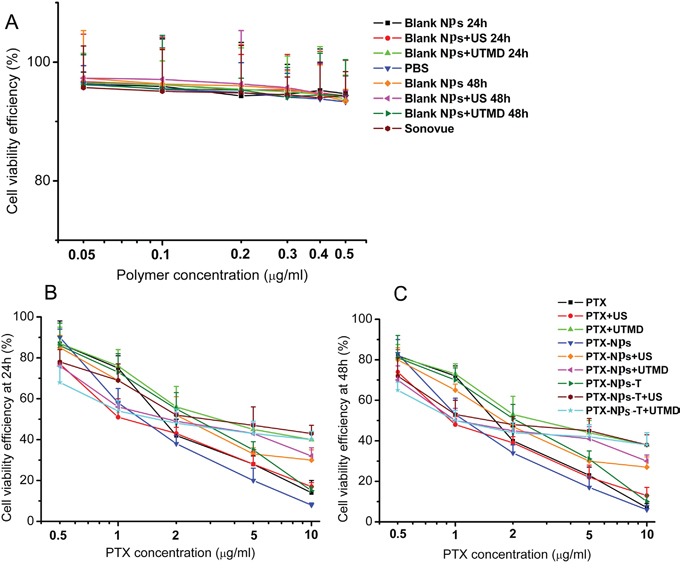
*In vitro* biocompatibility and cytotoxicity of NPs **A.** Toxicity of blank NPs to AFPC-1 cells. **B.** Toxicity of PTX-NPs to AFPC-1cells at 24h. **C.** Toxicity of PTX-NPs to AFPC-1cells at 48h.

The cell viabilities of AFPC-1 cells following administration of PBS, SonoVue, blank NPs, US, or UTMD for 24 or 48 h were all between 92 and 98% (Figure 6A). No significant differences in cell viability were identified between these groups (*p*>0.05).

Under the Optimal US and UTMD conditions, it was possible for more targeted NPs to ingress the cells, guaranteeing the cytotoxic effect *(p< 0.05*). US and UTMD might promote NPs to enter the pancreatic cancer cells, more mechanisms might be unclear besides sonoporation. The efficiencies of US and UTMD to enhance NPs into pancreatic cancer cells were enhanced related to saturation of holes on the surface of cell membranes (Figure 6B, 6C).

### Two-dimensional ultrasonic imaging and ultrasound contrast enhanced imaging of UCAs *in vitro*

**Figure 7:**
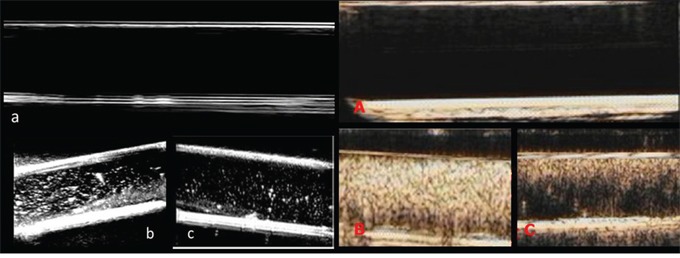
Two-dimensional ultrasonic imaging and ultrasound contrast enhanced imaging of UCAs *in vitro* Two-dimensional ultrasonic images: **a.** a tube filled with degassed aqueous, **b.** SonoVue in degassed aqueous. **c.** NPs in degassed aqueous. Ultrasound contrast enhanced images: **A.** a tube filled with degassed aqueous, **B.** SonoVue in degassed aqueous. **C.** NPs in degassed aqueous.

Echoless was showed in the degassed aqueous neither in two-dimetional ultrasonic imaging nor in contrast enhanced imaging. NPs and SonoVue both showed dotted–like echoes in two-dimetional ultrasonic imaging and in contrast enhanced imaging. The difference was the dotted-like echoes of NPs were smaller than those of SonoVue (Figure 7).

### Two-dimensional ultrasonic imaging and ultrasound contrast enhanced imaging of UCAs *in vivo*

**Figure 8:**
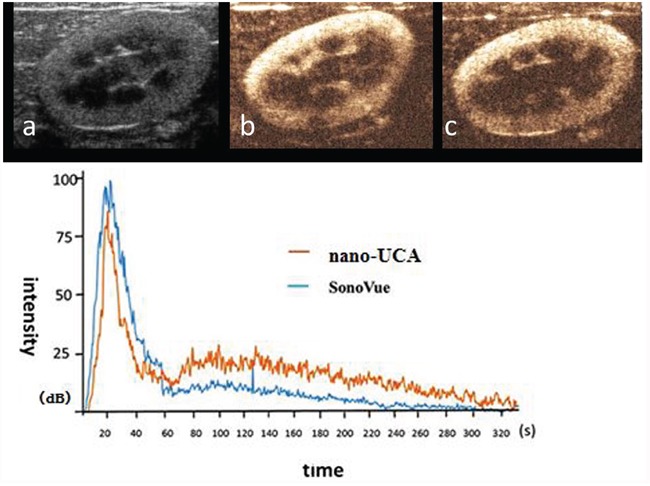
Two-dimensional ultrasonic imaging and ultrasound contrast enhanced imaging of UCAs in rabbit kidney and the time vs. intensity curve **a.** two-dimensional ultrasonic images of rabbit kidney, **b.** SonoVue US contrast enhanced image of rabbit kidney at the filling peak **c.** NPs ultrasound contrast enhanced image of rabbit kidney at the filling peak.

Dynamic ultrasound contrast imaging process of rabbit kidney: To the kidney of rabbit, at the fifth second after vein bolus-injection, NPs began filling with star-shaped, at the 24^th^ second reached to peak, last for six seconds, at the 30^th^ second, began pulling out quickly; at the 48^th^ second, began pulling out slowly, cleared up completely until 8 minutes. Correspondingly, at the 3^rd^ second after vein bolus-injection, SonoVue began filling rapidly, peaked at the 28^th^ second, lasted for 8 seconds, then faded swiftly, began fading slowly at 48^th^ second, until 5 min, dissipated entirely. The SonoVue filling peak was higher than NPs, but the filling peak time of the nanoparticles was ahead of that of SonoVue, the pulling-out time of NPs lasted for longer than SonoVue (Figure 8).

**Figure 9:**
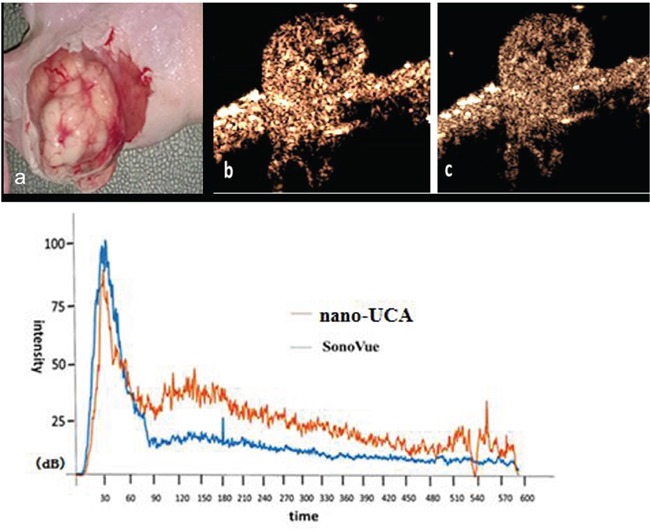
Ultrasound contrast enhanced imaging of two kinds of UCAs in superficial implantation tumor loaded mice and the time vs. intensity curve **a.** superficial implantation tumor of pancreatic cancer in nude mouse. **b.** SonoVue ultrasound contrast enhanced imaging of superficial implantation tumor of pancreatic cancer in nude mouse at the filling peak. **c.** NPs ultrasound contrast enhanced imaging of superficial implantation tumors of pancreatic cancer in nude mouse at the filling peak time.

Dynamic ultrasound contrast enhanced imaging process of two kinds of UCAs to the pancreas superficial implantation tumor was observed. At the 8^th^ second after vein bolus-injection, NPs quickly filled the tumor from the peripheral to the center with dendritic-shaped. At the 33th second, the imaging strength reached peak and lasted for 35 seconds. The strength subsequently pulled out slowly and extinguished completely in 10 minutes. Correspondingly, SonoVue filled rapidly at the 5^th^ second after vein bolus-injection, reached peak at the 35^th^ second, then faded in 9 minutes. The SonoVue peak was higher than nano-UCA, but nanoparticles lasted longer than SonoVue. (Figure 9).

**Figure 10:**
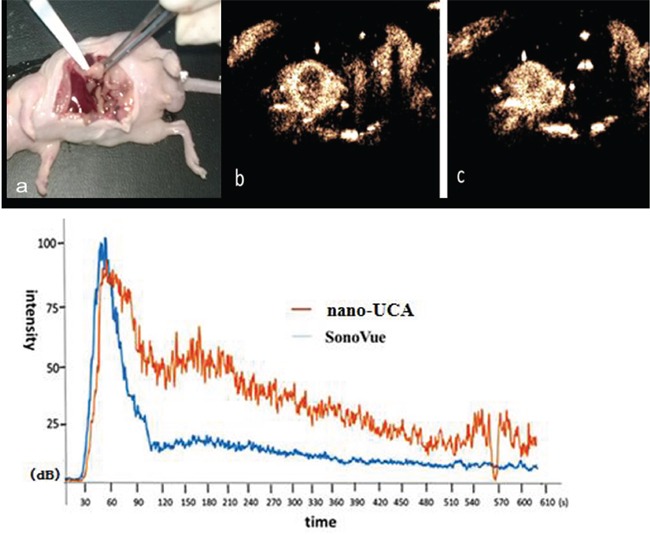
Ultrasound contrast enhanced imaging of UCAs in orthotopic implantation tumor of pancreatic cancer in nude mice and the time vs. intensity curve **a.** Orthotopic implantation tumor of pancreatic cancer in nude mouse, **b.** SonoVue ultrasound contrast enhanced image of orthotopic implantation tumor of pancreatic cancer in nude mouse at the filling peak time, **c.** NPs ultrasound contrast enhanced image of orthotopic implantation tumor of pancreatic cancer in nude mouse at the filling peak time.

Dynamic ultrasound contrast enhanced imaging process of the pancreas orthotopic implantation tumor was observed. At the 33th second after vein bolus-injection, NPs filled contemporaneously to the total tumor. At the 60^th^ second the imaging strength reached peak and lasted for 80 seconds. Subsequently, it pulled out slowly in 10min. At the 38^th^ second after vein bolus-injection, SonoVue filled the total tumor. At the 70^th^ second, the imaging strength reached peak and pulled out quickly in 10min. The pulling out time of NPs was longer than SonoVue. (Figure 10).

## DISCUSSION

Macromolecular substances like NPs may access the cells through the mechanism of their endocytosis under the condition of no US and UTMD. Correspondingly, some possible impacting mechanisms of US or UTMD have been reported as the micro-circumflex, micro-fluid actions and so on may punch transient recoverable holes in their surfaces to motivate NPs into the cells [16–20], though the genuine mechanism remains unclear. Our research team previously proposed a novel possible mechanism, which was UTMD stimulate grid protein to promote cellular endocytosis and detailed statements were as follows: on one side, cells transportation consistently exists for a long time after the action of US or UTMD, rather than gradually weakens, for another, enhanced peak of the cells uptake was oriented at the time of the administration of UTMD 45min later, when cellular clathrin-dependent endocytosis time was overlapped [21]. The reason might be because of the suitable intensity of mPEG-PLGA. When the ultrasound is added on SonoVue, the liposome deforms instantly and strongly to good harmonic image. While the porous nanoparticle of mPEG-PLGA material may have a litter harder shell to deform weakly, possibly becoming stronger according to its accumulation intensity gradually. However, it was difficult for NPs (100 nm or so) to access the nucleus owing to the effective pore size (only 9 nm or so) of nucleus membrane [22]. Besides, the action power of the optimal US or UTMD was safe and impossible to affect the nucleus membranes [23, 24].

In brief, firstly, the ultrasound contrast enhanced images were good enough to show lesions in terms of this kind of novel targeted NPs. The hollow holes in lyophilized multi-porous targeted NPs may be so small that stronger ultrasonic reflection and scattering signals would require a higher concentration of the targeted NPs aqueous for US contrast imaging [25, 26], compared with SonoVue. Though no obvious difference was detected at the beginning or peak time of the two kinds of UCAs, targeted NPs faded more slowly in comparison with SonoVue. The longer clear-time of targeted NPs lasted for 10 min, which demonstrated that in one respect, it was very possible for them to enter more microvasculars than SonoVue. For another, targeted NPs could partly penetrate into organization gaps and bind to the surface of tumor cells to form aggregation effect.

Conventional UCAs such as lipid-shelled SonoVue which was filled with sulfur hexafluoride gas have been widely used in clinic work. These types of UCAs have the function of excellent contrast enhanced imaging. However, they cannot give some corresponding therapy in the meanwhile. The double-targeted NPs could not only supply contrast enhanced imaging but also deliver drug into the tumor cells. It was a promising preparation in clinic. There are still some efforts should be done in our future work. The particle size should be reduced to improve the imaging efficiency. And the drug load ratio should be further improved to enhance tumor inhibition effect.

## MATERIALS AND METHODS

### Materials

The 1-(3-Dimethylaminopropyl)-3-ethylcarb odiimide hydrochloride (EDC), N-hydroxysuccinimide (NHS), rhodamine (Rh) and fluorescein isothiocyanate isomer (FITC) were purchased from Sigma Aldrich Company (Shanghai, China). The mPEG-PLGA (mPEG 5000, GA: LA=8:2) was donated by the Shanghai Cancer Institute (Shanghai, China). Dialysis bags were purchased from Lv Niao technology Co. Ltd (Shanghai, China). Paclitaxel was from Jiangsu yew pharmaceutical Co. Ltd (Jiangsu, China). Pluronic-F68 (F68) was obtained from BASF (China) Co. Ltd. (Shanghai, China). CCK-8 kit was purchased from Dong Ren chemistry Co. Ltd (Shanghai, China). IMDM medium and 10% fetal bovine serum, penicillin, streptomycin were purchased from Gibco (New York, USA). CA19-9 antibody, CEA antibody and 4, 6-diamino-2-phenyl indole (DAPI) were purchased from Qian Chen Biological Technology Co.Ltd (Shanghai, China). Other reagents were from Sinopharm Chemical Reagent Co.Ltd (Shanghai, China).

The CFAPC-1 pancreatic cancer cells were purchased from Cell Bank, Shanghai Institutes for Biological Sciences, Chinese Academy of Sciences (Shanghai, China). SonoVue was from Bracco Imaging. BV (Milan, Italy). The animals were supplied by the First People' Hospital Affiliated to Shanghai Jiao tong University (Shanghai, China). All animal procedures were performed according to the research protocol approved by the Animal Care and Use Committee at the First People' Hospital Affiliated to Shanghai Jiao tong University.

### Fabrication of PTX-mPEG-PLGA NPs

Firstly, 3.5mg PTX and 125mg mPEG-PLGA were dissolved into 5ml dichloromethane solution. Then 200uL 0.5mg/ml F68 solution was added into the dichloromethane solution and emulsified for 1.5 min by the work-frequency (one time per 2second). Subsequently 50ml F68 solution was added and emulsified for 1min by the same work-frequency mentioned. The dichloromethane was removed by stirred at room temperature. The NPs was lyophilized to remove the inner water phase.

### Fabrication of the double-targeted PTX-NPs modified by anti-CEA antibody and anti-CA19-9 antibody

50mg PTX-mPEG-PLGA was dispersed into 50ml water and 1mg CA19-9 and 1mg CEA was added. Then, 1mg EDC and 1mg NHS were dissolved into the solution and stirred for an hour at room temperature. The solution was dialyzed (molecular weight cut off (MwCO=3500) for an hour to remove EDC and NHS. The NPs solution was then separated through a sephadex G50 column to remove the unconjugated antibodies.

### Characterization of the double-targeted PTX NPs

The size distribution and zeta potential of the double-targeted NPs were determined using a particle size/zeta potential analyzer from particle sizing system, Inc. (Florida, USA). The morphology characteristics of the double-targeted PTX-mPEG-PLGA NPs were observed using transmission electronmicroscopy (TEM) from Hitachi, Ltd. (Japan). The infrared spectrum of double-targeted PTX-mPEG-PLGA NPs were detected by Vertex 70FT-IR Spectrometers (Germany).

We observed the double-targeted NPs compared to SonoVue under optical microscope. 20ul NPs degassed aqueous (mPEG-PLGA concentration: 17mg/ml) and 20ul SonoVue degassed aqueous (concentration: 17mg/ml) were respectively observed with optical microscope.

The drug entrapment efficiency of PTX was determined by the high performance liquid chromatograph (HPLC). 200ul NPs water solution was added into 2ml volumetric flask and dissolved with acetonitrile. The NPs were dissolved by ultrasonic and filtered with 0.45μm microfiltration membrane and detected by HPLC analyzer. The detection parameters were as follows: Hypersil BDS C18 chromatographic column (Dalian elite Co. 150×4.6mm, 5μm). Acetonitrile-10mmol/L NH_4_Ac solution (pH 5.0) 53:47 was as the mobile phase. The column temperature was 30°C. The flow rate was 1ml/min. The detection wave was 227nm.

The entrapment efficiency was defined by the ratio of the amount of PTX embedded in the double-targeted PTX-mPEG-PLGA NPs to the total amount of PTX incipiently used. The loading drug efficiency was defined by the ratio of the weight of PTX embedded in the double-targeted PTX-mPEG-PLGA NPs to the weight of double-targeted PTX-mPEG-PLGA NPs.

### Drug release of PTX from the double-targeted NPs

A dialysis-bag (MWCO=3500), in which 1ml PTX-mPEG-PLGA NPs aqueous was sealed, was immersed into 19ml phosphate buffered saline (PBS) solution containing 1moL/L sodium salicylate (pH=5.0, 6.5, 7.4) and shaked at 37°C. At predetermined time, 200 μL sample of the dialysis bag were taken out and fresh medium of the same volume was added. PTX dimethylsulfoxide (DMSO) solution was used as control. PTX concentrations of samples were determined by HPLC and release quantity of PTX was calculated to draw the cumulative release-time curves. PTX release efficiency was determined by the ratio of the PTX amount released into solution to the total amount of PTX in the double-targeted NPs.

### US and UTMD condition optimization

Human Pancreatic cancer CFAPC-1 cells were incubated in Iscove's Modified Dulbecco's Medium (IMDM) with 10% fetal bovine serum (FBS), penicillin and streptomycin (100 μg/ml) at 37°C in a humidified condition with 5% CO_2_.

A therapeutic US machine (Physiomed, Erlangen, Germany) was used, on which some conditions were fixed as followed: the frequency of the probe was 1MHz, duty cycle:1:5. According to US power, US irradiation time, SonoVue volume ratio, diverse experiment conditions were designed into 20 groups (Chart 1). Encapsulating RhB double-targeted NPs were added into CFAPC-1 cells and incubated for 2hr under the 20 setting conditions respectively. Then the cells were trypsinized and collected to be analyzed by flowcytometry (FCM).

**Chart 1:** US and UTMD conditions

Group	US power	US irradiation time	SonoVue volume ratio
1	0.8w	40s	20%
2	0.8w	60s	20%
3	0.8w	80s	20%
4	1.0w	40s	20%
5	1.0w	60s	20%
6	1.0w	80s	20%
7	1.2w	40s	20%
8	1.2w	60s	20%
9	1.2w	80s	20%
10	No US		
11	0.8w	40s	40%
12	0.8w	60s	40%
13	0.8w	80s	40%
14	1.0w	40s	40%
15	1.0w	60s	40%
16	1.0w	80s	40%
17	1.2w	40s	40%
18	1.2w	60s	40%
19	1.2w	80s	40%
20		RhB solution, no US	

### Cell uptake observed by confocal laser scanning microscope (CLSM)

CFAPC-1 cells were incubated in confocal dishs for 2 hrs with RbB-mPEG-PLGA NPs and RbB-double-targeted NPs respectively. The US or UTMD condition was optimized above. The cells were fixed and dyed before viewed through CLSM.

### Cyto-compatibility and antitumor effect of double-targeted NPs

The cellular cytotoxicity of the NPs was determined by MTT assay. CFAPC-1 cells (1×10^5^/well) were cultured in 96-well plates for 24 h. Then the cells were treated with different conditions and incubated for 24 and 48 h at 37°C. The fixed probe frequency was 1 MHz, duty cycle was 20%. The optimal US (power, 1 W/cm2; exposure time, 60 sec) or UTMD conditions (power, 1 W/cm2; exposure time, 60 sec; SonoVue volume ratio, 2:5) were used. Subsequently, the medium in the wells were removed and 0.2 ml fresh medium of MTT (0.5 mg/ml) was added to every well and incubated for 4 h at 37°C. The culture medium was then removed from the wells and replaced with 0.2 ml dimethyl sulfoxide. Following agitation of the 96-well plates for 15-20 min, the absorbance was measured at a wavelength of 490 nm using a Model 680 Microplate Reader from Bio-Rad Laboratories (Hercules, CA, USA).

### Respectively two dimensional ultrasound and ultrasound contrast enhanced imaging of NPs *in vitro*

Philips IE33 was used and the probe was L11-3. Firstly, NPs powder were respectively put into Eppendorf tube (concentration: 17mg/ml) filled with degassed water, sealed and vibrated fully. The outer surfaces of the tube were covered with ultrasound coupling agent to keep no air between the tubes and the transducer. Ultrasound contrast enhanced images was taken immediately and SonoVue was observed as comparison.

### Respectively two dimensional ultrasound and ultrasound contrast enhanced imaging of NPs and SonoVue *in vivo*

The CFAPC-1 orthotopic tumor was set up by injecting 20μl CFAPC-1 cells spension (5×10^7^/ml) into the pancreas of BALB/c nude mouse.

Siemens Sequaio512 and LOGIQ E9 were used and the corresponding probes were respectively 15L8W-S and ML6-14. SonoVue degassed aqueous(0.2 ml, concentration: 17 mg/ml) and NPs degassed aqueous (0.2 ml, concentration: 17 mg/ml) were quickly injected into rabbit ear veins respectively, in the meanwhile, ultrasound contrast imagines of the rabbit right kidney were real-time observed and recorded. In the same way, 0.1 ml NPs and 0.1 ml SonoVue were quickly injected into tail vein of orthotropic pancreas tumors loaded nude mice respectively. At the same time, ultrasound contrast enhanced imaging of superficial was also real-time observed and recorded.

### Statistics

Measurement data were displayed with x^−^±sd. Statistical tests were processed using the Student's *t*-test, and the statistical difference for significance between the experimental and control groups was analyzed using SPSS19.0 software. The statistical significance was determined at *p* values < 0.05.
